# Occupational therapy practice for post‐acute COVID‐19 inpatients requiring rehabilitation

**DOI:** 10.1111/1440-1630.12976

**Published:** 2024-06-14

**Authors:** Hayley M. Scott, Sharon Neale, Elizabeth Harrington, Hayley Hodgson, Danielle Hitch

**Affiliations:** ^1^ Occupational Therapy Lecturer, Institute of Health and Well‐Being Federation University Melbourne Australia; ^2^ Occupational Therapy Department Western Health Melbourne Australia; ^3^ School of Health and Social Development, Deakin University and Occupational Therapy Department Western Health Melbourne Australia

**Keywords:** COVID‐19, inpatient, occupational therapy, rehabilitation, SARS‐CoV‐2

## Abstract

**Introduction:**

COVID‐19 caused significant occupational disruption to people's life roles, with some people requiring an inpatient rehabilitation admission. Occupational therapists assessed and treated these patients using previous knowledge of similar conditions due to limited specificity in available guidelines to inform practice. The aim of this study was to investigate current practice with post‐acute COVID‐19 (PAC) patients within an inpatient rehabilitation setting in Australia, to better understand the role and impact of occupational therapy.

**Methods:**

A mixed‐method study was conducted, including electronic medical record audits (October 2021 October 2022) and descriptive patient interviews at a large metropolitan subacute service. Descriptive statistics and qualitative analysis were used to summarise and interpret data.

**Consumer and Community Involvement:**

No involvement.

**Results:**

A total of 24 patient electronic medical records were audited, and 10 patient interviews were completed. Three overarching categories were identified within the 685 occasions of occupational therapy service audited—occupational engagement, education provision and discharge planning. Patients identified the value of occupational therapy by reflecting on their lived experiences of engaging with occupational therapists and associated changes in occupational performance between COVID‐19 diagnoses and discharge home.

**Conclusion:**

Occupational therapists possess a unique skill set that directly addresses the occupational needs and priorities of PAC patients. This study adds to the growing body of evidence supporting the contribution of occupational therapy to the management of COVID‐19; however, further research is needed to develop evidence‐based practice resources and advocate for system changes that improve quality of life for COVID‐19 patients.

**PLAIN LANGUAGE SUMMARY:**

During the COVID‐19 pandemic, a lot of people got very sick. Some of these people needed more time and support to get better. Occupational therapists were important during this time because they helped these people to do their daily activities again. Because there were not many resources on how to do this, we looked into what occupational therapists were doing to help these people. We looked at patient hospital files and also talked to them to understand this better. We found that occupational therapists focused on three main areas: helping patients do activities that were important to them, teaching them about COVID‐19 and helping them plan to leave the hospital. This study shows that occupational therapists are skilled at helping people with COVID‐19. But more research is needed to make resources and also help with changing the healthcare system to further help people get better from COVID‐19.

**Key Points for Occupational Therapy**

- Patients value the broad range of occupational therapy interventions which addressed their individualised needs and preferences.
- Inpatient occupational therapists mostly commonly provided interventions for personal care occupations.
- Patients value the opportunity to practice meaningful occupations to improve their confidence and self‐esteem.

## 1 INTRODUCTION

The COVID‐19 pandemic has posed unprecedented challenges to global healthcare systems. Despite relatively low case numbers initially and high levels of vaccination, people in Australia have not escaped the profound effects of the pandemic. As of 22 September 2023, Australia had recorded 11,591,525 cases and 22,887 deaths from COVID‐19 since the beginning of the pandemic (World Health Organisation, 2023a). While some of these cases are reinfections, these numbers are likely underestimated given that reporting of polymerase chain reaction (PCR) or rapid antigen test (RAT) results are no longer mandatory in many workplaces, schools or for travel purposes (State Government of Victoria, 2022).

Whereas most people make a full recovery from their COVID‐19 infection, a noticeable minority experienced sustained and significant disability. Long COVID (also known as post‐acute COVID condition [PACC]) was first identified by patients in May 2020 (Callard & Perego, 2021). The World Health Organisation (WHO) developed a case description of Long COVID as “the continuation or development of new symptoms 3 months after the initial SARS‐CoV‐2 infection, with these symptoms lasting for at least 2 months with no other explanation” (WHO, 2021, p.1). However, the Centres for Disease Control and Prevention (Centres for Disease Control and Prevention, 2023) recognises Long COVID in people experiencing ongoing symptoms which significantly impact on function and quality of life in the medium term (4 to 12 weeks post infection). Variations in definitions and a lack of diagnostic criteria for this syndrome are ongoing challenges for both healthcare providers and patients. In this paper, we use the term Post‐Acute COVID‐19 (PAC) in recognition that the patients in this study were experiencing difficulties stemming from an acute COVID‐19 infection but may or may not have met the criteria for Long COVID.

The range of sustained symptoms and functional problems reported following COVID‐19 infection are strikingly diverse. Commonly reported symptoms include fatigue, breathlessness, smell and taste dysfunction, myalgia and cough (Healey et al., 2022). A cross‐sectional study from Denmark (Nielsen et al., 2022) described the functional problems experienced by patients (*n* = 448) attending a post COVID‐19 outpatient clinic. Around three quarters of the sample experienced moderate to severe mental fatigue, and most patients experienced significant difficulties completing productive and leisure activities. Over half of the patients (56%) were also on sick leave from their usual employment. Most recently, a cohort study of over 6 million Americans (Bowe et al., 2023) found that Long COVID contributed 80.4 disability adjusted life years per 1000 persons, indicating a higher level of disability than cancer or heart disease.

Australians recovering from COVID‐19 have also described the widespread impact of this syndrome in their daily life, describing recovery as getting back to ‘their normal’ (Hitch et al., 2023). Over time, it became increasingly evident that many PAC patients require support for rehabilitation and recovery. The final report of an inquiry into Long COVID (Parliament of Australia, 2023) highlighted the need for early access to individualised rehabilitation services for people experiencing functional impairment following COVID‐19 infection. The WHO (2023a) living guideline for the clinical management of COVID‐19 recognises occupational therapists as integral members of the rehabilitation workforce for PAC patients particularly regarding their expertise in multimorbidity and functional decline.

An evidence base for rehabilitation for PAC patients is only now emerging and suggests that tailored multidisciplinary rehabilitation programs are an effective approach to maximise both short‐ and medium‐term outcomes (Grishechkina et al., 2023). Studies of the impact of allied health for PAC patients receiving inpatient rehabilitation (Bellinger et al., 2021) and a scoping review of rehabilitation programs for older adults post PAC hospitalisation (Araya‐Quintanilla et al., 2023) both mention occupational therapy as part of the multidisciplinary team approach. To date, limited research has specifically focused on occupational therapy practice, yet some successful interventions have been recognised. A case study from the USA (Wilcox & Frank, 2021) described occupational therapy assessment and intervention with a patient which included functional endurance assessments, time use, occupational balance interventions, education on symptom self‐management and a multicontext approach to functional cognition. A study by Swiss occupational therapists found that patients experienced anxiety and reduced self‐perception; however, a feasible Energy Management Education intervention was developed that showed positive effects on occupational performance, self‐efficacy and quality of life (Hersche & Weise, 2022). A more recent study of the Stop‐Rest‐Pace approach (Tanguay et al., 2023) delivered by occupational therapists and physiotherapists found up to two‐thirds of patients continued to experience post exertional malaise after receiving this intervention. Establishment of an evidence base about how occupational therapy supports PAC patients is essential to demonstrate the profession's distinct and meaningful impact for this patient cohort. Emphasising the unique value of occupational therapy is also critical to establishing the profession's presence in the services, systems and policies currently being developed for this growing cohort of patients.

Despite these mentions of occupational therapy in the international literature, the Australian evidence‐based clinical guidelines (National Covid‐19 Clinical Evidence Taskforce, 2023) do not specifically refer to the profession. This is likely due to there being no Australian studies of the occupational therapy role with PAC patients. The development of clinical guidelines for the management of PAC patients requires an evidence base to inform detailed guidance on discipline‐specific assessments and interventions for a patient cohort, which is currently lacking. Additionally, there is a greater need for local evidence due to the unique context of our geography and healthcare systems, and the impact of national and state‐based policy responses to the pandemic. The need for local research can be highlighted by a study which found that many PAC patients seeing occupational therapists in overseas settings were initially referred for assessment of dysphagia (Christensen et al., 2022; von Zweck et al., 2023), which is beyond the scope of occupational therapy in Australia.

Therefore, the aim of this study was to investigate current practice with PAC patients within an inpatient rehabilitation (IPR) setting in Australia to better understand the role and impact of occupational therapy to help inform clinical guideline development. The research questions were as follows: (i) What are the characteristics of PAC patients requiring inpatient rehabilitation with regards to clinical frailty, length of stay, medical complications, vaccination status, functional outcomes and discharge destination?; (ii) What is current occupational therapy practice for PAC patients in inpatient rehabilitation?; (iii) From the patients perspective, what impact does inpatient rehabilitation occupational therapy have on their recovery?

## 2 METHOD

### 2.1 Study design

This study adopted a descriptive mixed‐method study design using a concurrent triangulation design (Creswell, 2014), collecting quantitative and qualitative data simultaneously and completing separate data analysis before comparing and contrasting the findings from each form of data. This pragmatic approach allows for flexibility within the methods, which provides insightful outcomes superior to either method used in isolation (Creswell, 2014). Retrospective electronic medical record (EMR) audits gathered quantitative data about patient characteristics and qualitative descriptions of occupational therapy interventions using an audit template. Patient interviews also captured qualitative data about patient perceptions and experiences of the impact of occupational therapy. For the purposes of this study, patient interviews consisted of patient experiences already collected by non‐treating clinicians which were utilised by the occupational therapy department for quality assurance. The study was approved by Western Health Human Research Ethics Committee (QA.2022.83_91736).) in December 2022 to retrospectively review EMR of these patients and their experiences.

### 2.2 Study setting

This study was completed on an adult IPR ward at a large metropolitan hospital in Victoria, Australia. The IPR ward consists of 30 beds providing multidisciplinary care to patients presenting with diverse medical conditions. While there were few PAC patients in the first year of the pandemic, there were increased admissions to address significant functional decline for this patient cohort during 2021.

### 2.3 Recruitment

#### 2.3.1 File audits

Using purposive sampling, all patients admitted to the IPR ward between October 2021 and October 2022 were screened for a primary diagnosis of COVID‐19 pneumonia and confirmed patients were entered into an Excel spreadsheet. The EMR of these patients was retrospectively audited over a 5‐week period during March–April 2023 as per ethics approval. Consent was not required as this data was already accessible as part of routine care. Due to the timing of the data collection, the study sample reflects COVID‐19 infections arising from the Delta and Omicron variants.

#### 2.3.2 Patient interviews

A non‐treating occupational therapist from the IPR ward was allocated to each patient. The non‐treating occupational therapist approached the patient, introduced the purpose and requested verbal consent to participate. Consenting patients were interviewed by the non‐treating occupational therapist 1–2 days before being discharged from hospital.

### 2.4 Data collection

#### 2.4.1 File audits

A bespoke audit tool collected the following information using the REDCap database (Vanderbilt University, 2023): (i) patient demographic information (i.e. gender, age, impairments, comorbidities, primary language), (ii) admission details (i.e. ICU admission required, ventilation requirements, vaccination status, length of stay, discharge destination, discharge follow‐up, primary impairments) and (iii) type and frequency of occupational therapy intervention provided, including clinical justification. See Appendix 1 for further details of audit tool template.

The primary outcome measure was the Functional Independence Measure (FIM) which was completed by nursing and allied health staff, as part of standard practice for patients on admission and discharge from the IPR ward. FIM scores were extracted from the EMR to reflect patient's functional changes over their admission. Additionally, the Clinical Frailty Score (CFS) was calculated, utilising the algorithm and information from the EMR. This was completed by non‐treating members of the research team to identify the frailty level of patients prior to their admission, adding to the patient demographic picture and overall fitness.

#### 2.4.2 Functional Independence Measure

The FIM is a standardised outcome measure designed to evaluate the functional abilities and progress of people undergoing rehabilitation (Uniform Data Set for Medical Rehabilitation, 2014). It includes 18 items across two domains: motor tasks (13 items) and cognitive tasks (5 items). These items quantify the person's level of independence in daily activities, which provides a total score ranging from 18 (lowest) to 126 (highest) level of independence. The FIM is a reliable and valid tool (Maritz et al., 2019), widely used in rehabilitation services across Australia; however, floor and ceiling effects of the FIM should be noted (Tokunga et al., 2017). FIM change scores of 17–22 points have been identified as a reasonable indicator of minimal clinically important change among other diagnostic groups (Arcolin et al., 2023).

#### 2.4.3 Clinical Frailty Score

The CFS (Rockwood et al., 2005) classifies patients according to nine levels of frailty, based upon activity and participation levels, with frailty defined as any score above 4 (Juma et al., 2016). The CFS was originally developed to measure frailty in outpatient clinical settings and has been shown to have very good inter‐rater reliability, predictive validity and clinicial utility (Gregorevic et al., 2016). To enhance reliability in the scale application, an algorithm developed by Chong et al. (2019) was provided to all researchers participating in data collection. This algorithm enabled excellent inter‐rater agreement and supported the predictive validity of the scale when used in an EMR audit of acute admissions for adults (Chong et al., 2019).

#### 2.4.4 Patient interviews

The interviews were conducted by four allocated occupational therapists in a private or semi‐private room on the IPR ward. Brief and informal training was provided, despite these therapists having experience in patient interviewing. Three specific questions were developed and piloted with similar patients prior to data collection, with no major changes suggested. These questions were as follows:

1. Can you tell me about your experience with doing your daily occupations since being diagnosed with COVID‐19?
2. Can you tell me about your experience working with occupational therapy during your rehabilitation stay?
3. You are leaving hospital soon; if you think back to when you first came to the rehabilitation ward to now the point of leaving, how are you feeling about participating in your daily occupations?

As the interviews were short in duration (5–15 minutes) and were collected on the IPR ward, it was not feasible to record them. Therefore, patient responses were written down, with key statements verbatim by the occupational therapist conducting the interviews reducing the burdensome on therapists during the COVID‐19 pandemic. Responses were then entered into a Microsoft Excel spreadsheet for analysis. Peer debriefing through supervision occurred for those therapists completing the patient interviews (Stanley, 2014).

### 2.5 Data analysis

#### 2.5.1 File audits

Retrospective EMR audit data was exported from the REDCap (Vanderbilt University, 2023) platform into an Excel spreadsheet for analysis. Simple summary statistics, reported as averages and percentages, were applied to describe patient demographics. Content analysis was used to describe occupational therapy practice which followed these steps: (i) preparation, (ii) organising and (iii) reporting the data (Elo & Kyngas, 2008). Occupational therapy intervention types were double coded by two independent clinician researchers using clinical judgement, and conclusions were discussed. Clinical judgements were also used where no clear clinical justification was provided in the file. Open coding and categories were identified from patterns in the data, before grouping codes under headings with general descriptions and final categories and subcategories were formed. Data was also triangulated using qualitative data regarding interventions that were added to the qualitative data from the patient interviews.

#### 2.5.2 Patient interviews

Content analysis was also used to analyse qualitative data from the patient interviews and EMR (Elo & Kyngas, 2008), which was a useful method given the short sections of text written by the occupational therapists (Stanley, 2014). One researcher team member transcribed patient responses from the interview sheets into an Excel spreadsheet. Two research team members familiarised responses by reviewing patterns of key words/phrases, which were then coded, collaboratively refined and allocated names to complete the analysis.

### 2.6 Positionality statement

With all research, it is helpful to understand our positionality and therefore our lens on the data. All authors are registered occupational therapists who are deeply committed to client‐centred care, aiming to inform evidence‐based, high‐quality care. Whereas the first four authors work clinically on the inpatient rehabilitation ward (some to lesser degrees), EH and HH were involved with data collection from patients not under their direct care. To minimise potential bias, HS and SN were involved in the data analysis. The last author, DH, possesses considerable qualitative research experience and expertise in the COVID‐19 domain, offering oversight and support within all aspects of the research.

## 3 RESULTS

### 3.1 File audits

#### 3.1.1 Characteristics of PAC patients

The EMR for 24 inpatient files were audited, with 10 of these patients participating in an interview. Participants ranged from 29 to 78 years of age (SD = 13.0), and 13 (54.2%) were female. Most patients were unvaccinated (*n =* 20, 83.3%), with 91.6% requiring an ICU admission. Whereas 21 patients (87.5%) were previously active and independent (as determined by CFS < 2), the PAC patients experienced various physical, cognitive and psychosocial impairments. Functional improvements were observed through an overall FIM change score of 31.09 (SD = 4.9), representing a minimal clinically importance difference. Most patients were discharged home (*n =* 23, 95.8%), and 18 (75.0%) patients had ongoing rehabilitation goals requiring referral to outpatient rehabilitation. Participants who were interviewed spent 16 days (SD = 12.8) longer on the rehabilitation ward. All participant characteristics are outlined in Table 1.

**TABLE 1:** Patient demographics from the file audits.

Characteristics		Not interviewed (*n =* 14)	Interviewed (*n =* 10)	Total (*n =* 24)
Age (years)		58 ± 14.7	57 ± 10.0	58 ± 13.0
Gender				
	Male	6 (42.8)	5 (50.0)	11 (45.8)
	Female	8 (57.2)	5 (50.0)	13 (54.2)
Language (English)		10 (71.4)	9 (90.0)	19 (79.1)
Unvaccinated		13 (92.8)	7 (70.0)	20 (83.3)
ICU admission		13 (92.8)	9 (90.0)	22 (91.6)
Intubation		9 (64.2)	7 (70.0)	16 (66.6)
Non‐invasive ventilation		3 (21.4)	1 (10.0)	4 (16.6)
No premorbid history		2 (14.2)	2 (20.0)	4 (16.6)
Clinical Frailty Scale ≤ 2		12 (85.7)	9 (90.0)	21 (87.5)
FIM score on admission	Total	73.6 ± 27.5	63.3 ± 22.4	69.6 ± 25.6
	Motor	44.8 ± 19.5	28.2 ± 17.3	38.3 ± 20.0
	Cognition	28.7 ± 8.7	29.5 ± 9.2	29.0 ± 8.7
FIM score on discharge	Total	103.1 ± 21.9	99.1 ± 13.2	101.5 ± 18.7
	Motor	72.0 ± 17.1	65.4 ± 12.9	69.4 ± 15.6
	Cognition	31.1 ± 7.2	33.6 ± 2.5	32.1 ± 5.9
Physical impairments				
	Muscular fatigue	11 (78.5)	8 (80.0)	19 (79.1)
	Oxygen requirements	7 (50.0)	8 (80.0)	15 (62.5)
	Shortness of breath	8 (57.1)	5 (50.0)	13 (54.1)
	Upper limb changes	6 (42.8)	5 (50.0)	11 (45.8)
	Pain	1 (7.1)	2 (20.0)	3 (12.5)
	Wounds	2 (14.2)	1 (10.0)	3 (12.5)
	Incontinence	1 (7.1)	1 (10.0)	2 (8.3)
	Other	1 (7.1)	1 (10.0)	2 (8.3)
Cognitive impairments				
	Confusion	5 (35.7)	2 (20.0)	7 (29.1)
	Memory	0 (0.0)	2 (20.0)	2 (8.3)
	Insight	1 (7.1)	1 (10.0)	2 (8.3)
	Attention	1 (7.1)	2 (20.0)	3 (12.5)
Psychosocial impairments				
	Anxiety	6 (42.8)	5 (50.0)	11 (45.8)
	Low mood	2 (14.2)	1 (10.0)	3 (12.5)
	Poor sleep	1 (7.1)	1 (10.0)	2 (8.3)
Length of stay				
	Acute	46.5 ± 36.1	81.7 ± 77.8	61.1 ± 58.4
	Inpatient rehabilitation	19.7 ± 16.6	35.9 ± 29.4	26.4 ± 23.6
Discharge destination				
	Home	13 (92.8)	10 (100.0)	23 (95.8)
	Residential aged care	1 (7.1)	0 (0.0)	1 (4.1)
Discharge follow‐up	ESD	0 (0.0)	1 (10.0)	1 (4.1)
	Rapid Allied Health	1 (7.1)	3 (30.0)	4 (16.7)
	CBR	8 (57.1)	5 (50.0)	13 (54.2)
	Other	5 (35.7)	1 (10.0)	6 (25.0)

Values are presented as mean ± standard deviation (SD) or number of participants (%).

Abbreviations: FIM, Functional Independence Measure; ICU, intensive care unit; ESD, early supported discharge; CBR, community‐based rehabilitation.

#### 3.1.2 Occupational therapy role in current practice

A total of 685 of face‐to‐face occasions of occupational therapy intervention were provided, with an average of 28.5 occasions per patient (SD 19.93, range 3–72). Three overarching categories were identified—occupational engagement (*n =* 378, 55.2%), education provision (*n =* 190, 27.7%) and discharge planning (*n =* 117, 17.1%). For each of these categories, codes were identified that highlighted what interventions were provided and the clinical justification for their use. A summary and tabulation of the interventions provided to PAC patients is provided in Table 2.

**TABLE 2:** Occupational therapy interventions.

Theme	Type of intervention	Total interventions (*n =* 685)
**Occupational engagement**		**378 (55.2)**
	Retraining within personal care occupations	109 (15.9)
	Mobility and transfer retraining within occupations	50 (7.2)
	Retraining within domestic occupations	45 (6.5)
	Energy conservation retraining within occupations	43 (6.2)
	Upper limb retraining	24 (3.5)
	Cognitive retraining	23 (3.5)
	Wheelchair retraining within occupations	22 (3.5)
	Motivational techniques within occupations	12 (1.7)
	Anxiety management within occupations	7 (1.0)
	Retraining in leisure occupations	5 (0.7)
	Retraining within community occupations Other—goal setting and review	3 (0.4) 35 (5.1)
**Education provision**		**190 (27.7)**
	Energy conservation education	65 (9.4)
	Use of oxygen within occupations	39 (5.6)
	Education to carers	19 (2.7)
	Rehabilitation expectations	15 (2.1)
	Equipment and home modifications	13 (1.8)
	NDIS eligibility	10 (1.4)
	Return to work/driving	7 (1.0)
	Anxiety management strategies	7 (1.0)
	Falls prevention	6 (0.8)
	Timetabling and routine within occupations	5 (0.7)
	Pressure care management	3 (0.4)
	Sleep hygiene	1 (0.1)
**Discharge planning**		**117 (17.1)**
	Communication with family, MDT and other stakeholders	35 (5.1)
	Equipment prescription	30 (4.3)
	Problem solving oxygen requirements at home	25 (3.6)
	Home assessments	10 (1.4)
	NDIS planning, documentation and implementation	7 (1.0)
	Complex equipment sourcing	6 (0.8)
	Carer training	4 (0.5)

Values are presented as number of occasions (%).

Abbreviations: NDIS, National Disability Insurance Scheme; MDT, multidisciplinary team.

#### 3.1.3 Occupational engagement

Patients had disengaged from meaningful occupations during their acute COVID‐19 infection, and occupational retraining was offered for domestic, leisure and community occupations one on one and within a group environment. However, personal care occupations were most often the focus of occupational therapy intervention. Patients experienced significant deconditioning, weakness and shortness of breath, necessitating a graded approach to interventions. Occupational therapists also utilised motivational techniques combined with deep breathing, to assist with building confidence and anxiety management during occupational engagement.

#### 3.1.4 Education provision

Occupational therapists provided various forms of education to patients, their family members and carers. Whereas patients were mostly educated on energy conservation techniques within occupations, occupational therapists also educated patients on management of new oxygen requirements at home and within the community. Other topics of education provision included falls prevention, use of equipment, pressure care, rehabilitation expectations, graded approach to recovery, sleep hygiene, return to work processes, and external follow‐up options such as accessing the National Disability Insurance Scheme (NDIS) for codiagnosis (i.e. acquired brain injury) post COVID‐19.

#### 3.1.5 Discharge planning

With most patients discharged home, common interventions provided by occupational therapists were gathering information of the home environment, conducting home assessments and recommending environmental interventions to facilitate discharge home. Environmental interventions consisted of alternative ways to manage oxygen requirements within the home, equipment prescription and setup. However, COVID‐19 restrictions imposed by the health service limited traditional face‐to‐face home assessments. Occupational therapists instead gathered information via the use of photos, measurements of the home environment taken by family or from online platforms and by completing virtual home assessments with family members. Other forms of intervention included carer training of mobility and transfers within the home environment and organisation of outpatient rehabilitation, support services and equipment supply to enable sustainable discharge.

### 3.2 Patient interviews

#### 3.2.1 Impact on occupational engagement and recovery

Three themes were formed. Findings are presented below using pseudonyms to protect the confidentiality of the patients.

##### Occupational challenges experienced by PAC patients

Patients reported experiencing a range of new feelings while completing occupations following their COVID‐19 diagnosis. Many reflected on the significant changes in occupational performance experienced due to increased difficulties completing simple occupations: “*It's been a big change … a typical day for me would be working full time, hanging out with my daughter and now I need assistance with all tasks*” *(Jordan)*.

Patients described the negative impact of their ongoing symptoms and recognised how much assistance they needed to participate in previously taken for granted occupations: “*I haven't been able to do anything really …*. *I'm flat out just trying to get to the toilet …. It was really debilitating because it was difficult to breathe. It was extremely hard … I really needed the help*” *(Charlie)*. These changes in function were often experienced as a complete disruption in their occupational lives: “*Getting dressed is no comparison; never had to learn how to eat again and now I need to think about everything … how much I can put in my mouth, how long I can brush my hair for … (it's been) a complete life change*” *(Peter)*.

A consistent feature of many descriptions of activity participation was the extra work and effort involved in occupational engagement: “*It's been really hard work, but I am pushing through. I get tired quickly … as doing things is harder. It's frustrating*” *(Georgie)*. These struggles also increased the time required to complete tasks, further exacerbating fatigue: “*It's a disaster; everything just takes so much longer and is more effort*” *(Ashley)*. The cumulative impact of functional difficulties was also evident, as described by ‘Sam’: “*Getting out of bed and my mobility were challenges that made everything else slow*”.

Feelings of fear, anxiety and being overwhelmed were commonly reported by patients during occupational engagement. ‘Taylor’ described how mental fatigue impacted on emotional responses, which were at odds with her usual approach to life:

“I had a level of fear doing my daily activities, however I'm really tired mentally … I'm a motivated person however initially my daily activities were so overwhelming; you know initially I couldn't even sit on the edge of the bed without being short of breath” (Taylor)

However, in some cases, this started to improve as they began to regain function: “*For a long time I needed to use a bed pan instead of going to the toilet. Over the last 3‐4 weeks I started to use the toilet and doing things more normally again. It was scary to begin with and more difficult to do things such as attending to my hygiene and going to the toilet*” *(Chris)*. Support from family appeared to be a facilitator in changing this mental state as described by ‘Ashley’: “*Having my wife allowed to be here has also helped me … .do more for myself which I like*”.

##### Helpful learnings and support from occupational therapy

When reflecting on experiences of working with occupational therapists during rehabilitation, patients identified various interventions they perceived as helpful. Many of these interventions helped the patient make sense of their overall recovery journey:

“The biggest thing I've learnt from OT is to pace myself. It's okay to sit and rest. It's a process. It'll take time and everyday you'll make small steps (which) really helped me to build my confidence ” (Taylor).

Patients received a range of interventions tailored to their specific needs, which included individual, group, face to face and online modalities. ‘Charlie’ explained how the diverse interventions he experienced contributed to his preparations for the future:

“The aids (long handled sponge and pick up stick) were helpful. The support was helpful in the breakfast club and thinking about how I need to be more organised and pace myself. The online group was also helpful to think of different ways to conserve energy. Very helpful in preparation to go home. It was helpful to prepare for what I might encounter and what to expect” (Charlie).

Patients appreciated the support, responsiveness and positivity demonstrated by their occupational therapists; “*Always present when needed, encouraging me to get back to doing things on my own. (Therapist) came home with me on the day of discharge to help setup equipment*” *(Jamie)*. The positive impact of the interpersonal interactions they had with occupational therapists on their motivation was mentioned by several patients, which supported their confidence to regain independence: “*Very helpful and friendly. Motivated me. Showed me that I can do things for myself*” *(Chris)*. The motivational support of the occupational therapists included targeted support around specific occupations and symptoms: “*Everyday making sure that I was up and dressed. (Therapist) has been so positive and supportive when I'm short of breath … I really appreciated it*” *(Ashley)*. A key aspect of these interactions was the professionalism that patients perceived from the occupational therapists, which enhanced their own feelings of confidence and ability: “*I had confidence in my OT, that you knew what you were talking about, which helped me improve my confidence to do my activities*” *(Taylor)*.

Patients also valued occupational therapy interventions that provided practical tips and strategies they readily applied to daily life. Despite the limitation of the inpatient setting, patients were provided with opportunities to practice various occupations in different forms: *“The energy group was great. I learnt a lot of strategies which I then practiced in the kitchen making my birthday cake*” *(Shannon)*. The use of both graded and compensatory approaches was frequently described, with these strategies used in a complementary manner: “*First sitting then I practiced standing in the shower. Getting down to the toilet was hard, took too long …. now I use a higher seat which makes life easier*” *(Sam)*. Occupational therapy was perceived as having a wide‐reaching impact on the patient's quality of life and recovery, as summarised by ‘Max’: “*(Therapist) taught me how to do everyday things, like putting my clothes on. It's been helpful. I didn't have an OT in acute but it has made such a difference*”.

##### The ongoing recovery journey

A key focus of occupational therapy on IPR is to prepare patients for safe and sustainable discharge. All patients described better confidence and improved occupational independence in the lead up to going home: “*I'm feeling good, much better than before. I wasn't able to do things like put my clothes on but now I'm feeling confident*” *(Ashley)*.

Whereas all members of the IPR multidisciplinary team contributed to their recovery and readiness for discharge, occupational therapists were perceived as the profession that focused on meaningful activities for patients: “*I now know what I need to do, to pace my daily activities, and I'm confident in doing this, despite needing oxygen still. What I learnt through the education and practice of doing things such as showering, going to the toilet, these are meaningful things which I do everyday*” *(Taylor)*.

Many patients recognised that their recovery would be ongoing and desired to continue working with the community rehabilitation service providers that their occupational therapists referred them to: “*Overall (I'm) much better but I still need more rehab so I can look at going back to work, cleaning my house and walking the dog*” *(Jamie)*. However, the prevailing perception was that discharge was an important and hard‐won milestone that provided a benchmark for reflecting on how far they had come: “*I never thought that I'd get out of here; such a big hill to climb. It impacted my whole life which changed (from) as always independent. I've been reborn and (now I) need to think and breathe*” *(Peter)*.

### 3.3 Combined analysis of EMR and interview data

**FIGURE 1:**
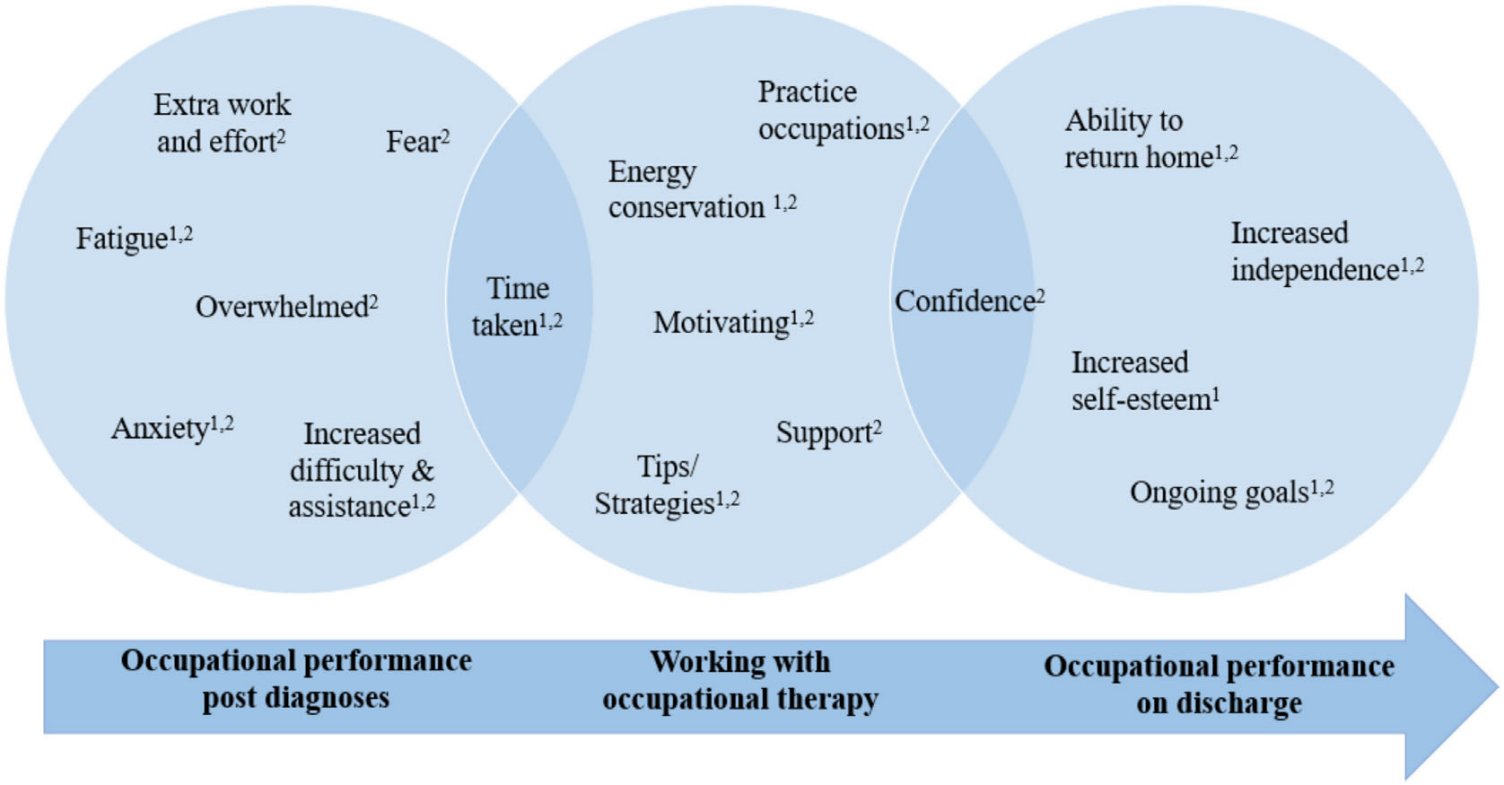
Occupational therapy intervention themes from EMR^1^ and patient experiences of COVID‐19 from patient interviews^2^.

As patients' occupational needs changed over time, so did the occupational therapy role. The qualitative experiences of PAC patients on IPR were reflective of the findings from the EMR audits. Interventions present in both the EMR and interview data are shown below in Figure 1. Firstly, patients reflected on their fatigue, altered breathing and anxiety and how simple occupations were now hard work. Secondly, patients recognised learning about new helpful tips and strategies such as pacing, use of aids/equipment and energy conservation techniques. Thirdly, patients valued the importance of being able to practice their daily occupations to help build their independence and confidence in preparation for discharge. These patient reflections are inclusive of occupational therapy practice categories of providing education, enabling occupational engagement and supporting discharge.

## 4 DISCUSSION

This study found that occupational therapists working in this IPR setting addressed occupational engagement, education provision and discharge planning for PAC patients. Majority of PAC patients were active and independent prior to their COVID‐19 infection, and were able to return home, despite a high proportion initially requiring ICU treatment. Muscular fatigue, shortness of breath, anxiety and confusion were the most common presenting symptoms. Patients valued occupational therapy interventions and reflected positively on their lived experience of engaging with occupational therapists, changes in occupational performance and being discharged home.

This is the first study to specifically investigate occupational therapy practice in the management of PAC patients within an Australian IPR setting. Recently, von Zweck et al. (2023) surveyed occupational therapists globally to describe the delivery and quality of occupational therapy interventions for people with COVID‐19 and post‐COVID‐19 conditions (PCCs). While Australian occupational therapists were likely included in the sample, international practices differ significantly such as dysphagia management being outside Australian scope of practice, which highlights the need for local health context research to promote generalisability to practice. Despite this, there were similarities regarding the most common delivery method of occupational therapy interventions which occurred in person, in contrast with providing remote interventions which occurred less frequently.

Findings of this study confirm that occupational therapists provide a broad range of interventions addressing the diverse symptoms and occupational performance issues experienced by PAC patients. Many of the interventions identified are consistent with international research which describe common occupational therapy interventions of fatigue management, cognition, relaxation, self‐management, environmental adaptation and mental health (von Zweck et al., 2023). Patients valued the broad range of interventions provided by occupational therapy that they perceived as addressing their personal needs and preferences. The positive statements from patients reflect broad themes outlined in the clinical practice guidelines like person‐centred care and management of oxygen requirements in daily activities (World Health Organisation, 2023b). Whereas specific interventions are not included in current clinical practice guidelines, occupational therapy practice in this study aligned with the guidelines' non‐specific COVID‐19 rehabilitation recommendations of ensuring that all patients are provided with education and support for self‐management of symptoms and resumption of daily activities, provide individualised rehabilitation programmes and that patients be screened for ongoing rehabilitation needs and referral prior to discharge (World Health Organisation, 2023b). Additionally, other research has reinforced and suggested further recommendations when working with COVID‐19 survivors, including supporting patients to remain connected with their community, minimising the functional impacts, providing integrated health as part of the multidisciplinary team, employing evidence‐based interventions, promoting patient education and improving the patient experience (Hitch & Holton, 2023; Ladds et al., 2020). Despite this, Australian occupational therapists report that these existing practice resources are insufficient, lacking specific and detailed guidance, and that new resources are needed to inform quality rehabilitation practices for these patients in the community (Van Laake & Hitch, 2024). In the absence of detailed guidelines, occupational therapists have relied on established interventions for similar conditions and presentations (Van Laake & Hitch, 2024). To build on these solid foundations, proactive efforts are needed to ensure that occupational research is included in the evolving clinical practice guidelines in this area. Further research and implementation are urgently needed to consolidate the occupational therapy role, evaluate the effectiveness of occupational therapy interventions and advocate for the unique contribution of the profession to recovery for PAC patients (von Zweck et al., 2023).

The findings of this study are also consistent with a Netherlands study (Nielsen et al., 2022), where most PAC patients receiving IPR were also active and independent prior to the onset of COVID‐19, which also caused significant loss in function and profound disruption to their lives. Similarly, inpatient interventions focused on building independence in occupations necessary for discharge home, such as personal care. Interventions that focused on engagement across the full range of meaningful occupations such as employment were addressed to a lesser extent due to time constraints. Returning to work has been consistently identified as a key aspect of recovery from COVID‐19 (Hitch et al., 2023; Hossain et al., 2023), recognising the potential impact on the person, their family and also the wider society due to economic factors. The role of occupational therapy with patients with PAC therefore extends far beyond the inpatient setting, particularly given that many people with Long COVID were not admitted to hospital in the acute phase of their infection (Malesevic et al., 2023).

However, access barriers to occupational therapy services for this cohort are a significant issue worldwide, attributed by limited funding, staff availability and insufficient resources (von Zweck et al., 2023). Nationally, many patients did not fit the referral criteria of community rehabilitation services during the pandemic due to significant waitlist pressure where only the most debilitated were prioritised (Parliament of Australia, 2023). Virtual community rehabilitation in the home environment is a feasible and acceptable modality for this patient group (Groenveld et al., 2022) potentially increasing service efficiencies. Further exploration of technology enabled home‐based rehabilitation and general advocacy around access to occupational therapy is also needed, to enable patients to achieve all occupational goals and promote sustainable health equity.

### 4.1 Limitations and future directions

This study was conducted during the COVID‐19 pandemic that caused significant limitations on the study. Despite our best efforts to overcome these challenges (as outlined below), the pandemic has affected the quality of the data. Firstly, it had a small sample size and was conducted at a single site due to ease of accessibility and limitations of COVID‐19 restrictions. Further studies should include a larger sample size, with the inclusion of multisites to validate these findings. Secondly, the study had no comparison group, and patient care was provided by a multidisciplinary team; therefore, the outcomes cannot be solely attributed to occupational therapy input. To mitigate these, patient interviews used were focused on occupational therapy input. However, patient interviews consisted of reuse of usual practice interviews capturing patient experiences and were not specifically designed for this study. Clinicians are very familiar with this process, offering expertise with capturing patients' experiences, and they used of a piloted set of questions for consistency; however, bias may be present as it was not feasible for the interviews to be audio recorded. The length of the interviews was also brief and varied between clinicians; however, this reduced the burdensome for participants and clinicians during a time of great strain on the health service. Further studies should include a more structured approach which includes audio recording and member checking to maximise rigour. Additionally, as all interviewed patients were discharged home, this may have influenced their positive experience. Thirdly, follow‐up after discharge was not performed. Therefore, it is not possible to confirm whether these outcomes persisted after discharge. A follow‐up evaluation is beneficial in confirming the long‐term effects and further journey of PAC patients. Despite these limitations, the mixed‐method approach provided timely rich data to understand the characteristics of this cohort and the occupational therapy role which can be used to inform future research.

## 5 CONCLUSION

Occupational therapists possess a unique and relevant skill set that addresses a broad range of needs and preferences for PAC patients. Occupational retraining, energy conservation, environmental adaptation and motivational guidance are already being applied in practice to increase patient independence, confidence and engagement in meaningful occupations. This comprehensive approach supports patients to return home after discharge; however, challenges may exist with community rehabilitation goals and optimal recovery due to systemic barriers to accessing occupational therapy in Australia. Further research is urgently needed to evaluate, strengthen and advocate for the occupational therapy role, in this rapidly changing and developing field.

## AUTHOR CONTRIBUTIONS

All authors meet the criteria for authorship. All authors were involved in the study design, analysis, interpretation of the data, drafting the work for intellectual content and final approval for publication. All authors acknowledge that the content has been reviewed and take responsibility for the data, analysis, interpretation and the conduct of the research.

## CONFLICT OF INTEREST STATEMENT

The authors have no conflict of interest to declare.

## Data Availability

The data that support the findings of this study are available on request from the corresponding author. The data are not publicly available due to privacy or ethical restrictions.

## Appendix

**Table:** RedCap audit template.

Audit heading	Audit response
Participant number	Numbers (1, 2, 3)
Gender	Male, female
Age	Open text
Date of hospital admission	Open text
Date of IPR admission	Open text
Date of IPR discharge	Open text
Acute admission LOS	Open text
IPR admission LOS	Open text
ICU admission	Yes, no
Ventilator	Ventilation, intubation, Non‐invasive, none
Vaccination status	Unvaccinated, 1, 2, 3, 4
Total LOS	Calculated tally
Reason for admission	Open text
Complications	Open text
Comorbidities	Open text
Primary language	Open text
CFS score	Numbers (1, 2, 3)
FIM motor admission	Open text
FIM cognition admission	Open text
FIM total admission	Open text
FIM motor discharge	Open text
FIM cognition discharge	Open text
FIM total discharge	Calculated tally
Discharge destination	Home, RACF, SDA, Transfer off ward
Discharge follow‐up	CBR, RAH, ESD, other
Primary impairments	Open text
OT intervention 1	
Initial assessment	Tick box
Goal setting	Tick box
Education—energy conservation (i.e. attendance to energy conservations group, provision of COVID booklet, 1:1 education)	Tick box
Education—time table to maximise occupation	Tick box
Education—return to work	Tick box
Education—return to driving	Tick box
Education—anxiety management (i.e. deep breathing, meditation, colouring, smiling minds, positive reinforcement)	Tick box
Education—sleep wake cycle	Tick box
Education—equipment and home environment	Tick box
Education—NDIS	Tick box
Education—current diagnosis/impairments	Tick box
Education—rehabilitation expectations	Tick box
Education—of use of oxygen within an occupation	Tick box
Education—pressure care	Tick box
Education—fall prevention	Tick box
Education—to family/care	Tick box
Home assessment—access visit	Tick box
Home assessment—virtual visit	Tick box
Home assessment—with patient	Tick box
Home environment—review in hospital (collecting photos, discussing with patient/carer)	Tick box
Home environment—problem solving occupational performance issues	Tick box
Equipment—prescription and education for home	Tick box
Equipment—prescription and education for the hospital	Tick box
Equipment—sourcing	Tick box
Retraining—motivational interventions (ie reassurance, validation, encouragement)	Tick box
Retraining—implementing energy conservation education within an occupation	Tick box
Retraining—personal occupations (including eating, grooming, shower and dress)	Tick box
Retraining—cognition	Tick box
Retraining—community occupations (road crossing, money management, shopping, etc.)	Tick box
Retraining—domestic occupations	Tick box
Retraining—anxiety management within occupations	Tick box
Retraining—shortness of breath, mobility and transfer	Tick box
Discharge planning/communicating with MDT/patient/family/carer/others	Tick box
NDIS documentation	Tick box
NDIS planning meeting	Tick box
Wheelchair assessment, retraining	Tick box
Other	Open text
OT intervention 2	
OT intervention 3 (continue for 30 occasions of intervention)	

Abbreviations: IPR, inpatient rehabilitation; LOS, length of stay; CFS, Clinical Frailty Scale; FIM, Functional Independence Measure; ICU, intensive care unit; ESD, early Supported discharge; CBR, community‐based rehabilitation; RACF, residential aged care facility; SDA, supported disability accommodation; NDIS, National Disability Insurance Scheme; OT, occupational therapy; MDT, multidisciplinary team.
